# Hybrid Ag–LiNbO_3_ nanocomposite thin films with tailorable optical properties†

**DOI:** 10.1039/d0na00975j

**Published:** 2020-12-28

**Authors:** Jijie Huang, Di Zhang, Zhimin Qi, Bruce Zhang, Haiyan Wang

**Affiliations:** School of Materials, Sun Yat-sen University Guangzhou Guangdong 510275 China huangjj83@mail.sysu.edu.cn; School of Materials Engineering, Purdue University West Lafayette IN 47907 USA hwang00@purdue.edu; School of Electrical and Computer Engineering, Purdue University West Lafayette IN 47907 USA

## Abstract

Ag nanostructures exhibit extraordinary optical properties, which are important for photonic device integration. Herein, we deposited Ag–LiNbO_3_ (LNO) nanocomposite thin films with Ag nanoparticles (NPs) embedded into the LNO matrix by the co-deposition of Ag and LNO using a pulsed laser deposition (PLD) method. The density and size of Ag NPs were tailored by varying the Ag composition. Low-density and high-density Ag–LNO nanocomposite thin films were deposited and their optical properties, such as transmittance spectra, ellipsometry measurement, as well as angle-dependent and polarization-resolved reflectivity spectra, were explored. The Ag–LNO films show surface plasmon resonance (SPR) in the visible range, tunable optical constants and optical anisotropy, which are critical for photonic device applications.

## Abstract 2

Ag nanostructures exhibit extraordinary optical properties, which are important for photonic device integration.

## Graphical abstract

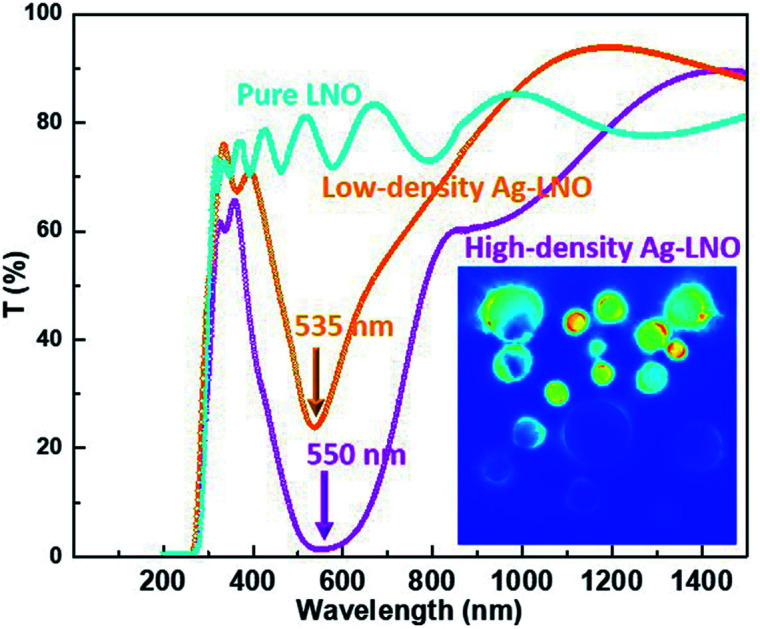

## Introduction

Nanocomposite thin films have attracted considerable research interest owing to their multifunctionality, heterointerface-induced physical phenomenon, strain engineering, as well as other promising applications in various fields.^1–4^ For example, multiferroic nanocomposite thin films have been achieved in multiple systems (*e.g.* BaTiO_3_ (BTO)–CoFe_2_O_4_ (CFO)^5^ and BiFeO_3_ (BFO)–CFO^6^), which are rarely obtained in single phase films. In addition, the exchange bias effect has been observed in ferromagnetic (FM)–antiferromagnetic (AFM) systems using the FM–AFM interface spin-orbital coupling such as La_0.7_Sr_0.3_MnO_3_ (LSMO)–NiO,^7^ BFO–Fe_3_O_4_,^8^ LSMO–LaFeO_3_ (LFO) and;^9^ previously, such an exchange bias effect was mostly achieved by a multilayer design. Furthermore, enhanced ferroelectricity has been realized in BTO–Sm_2_O_3_ ^10^ by lattice mismatch-induced strain. Taking advantage of charming properties of nanocomposite thin films, various devices could be developed for desirable performance, including nonvolatile switching devices^11^ and magneto-resistive devices.^12^

In another side, because of its unique optical and electrical properties, the Ag nanostructure has been extensively studied in the past few decades. The potential applications of Ag nanostructure vary from bio-related applications (*e.g.* bio-sensing, antibacterial)^13–15^ to energy-related applications (*e.g.* photoelectric, catalysis).^16–18^ Currently, most studies have focused on the synthesis approach and morphological regulation of Ag nanostructures. The synthesis methods, such as chemical reduction,^19^ polyol process,^20^ laser ablation in aqueous solution,^21^ and an electrochemical method, are mostly solution-based.^22^ The performance of the Ag nanostructure largely depends on its size, density (distribution), shape (nanosphere, nanocube, nanowire, and nanotriangle) and surrounding medium.^23–26^

In this study, we employed the concept of the nanocomposite thin film to fabricate Ag nanoparticles (NPs) into LiNbO_3_ (LNO) to form a Ag–LNO nanocomposite thin film. Recently, metal–oxide nanocomposite thin films have been extensively explored, and extraordinary physical properties, such as magnetic anisotropy,^27,28^ plasmonic resonance,^29^ and hyperbolic property,^30^ have been obtained. Interestingly, the shape of the metal nanostructure highly depends on the oxide matrix, *e.g.*, Au nanorods exist in the Au–BTO system,^29^ while Au NPs form in the cases of Au–LNO,^31^ Au–VO_2_ ^32^ and Au–LiNi_0.5_Mn_0.3_Co_0.2_O_2_.^33^ Furthermore, the growth mechanism of the metal–oxide system is indeed complex and currently under investigation. It involves sophisticated kinetics and thermodynamics processes, in addition to crystal lattice matching. Thus, additional investigation is required to completely understand the growth mechanism of metal–oxide nanocomposites. Here, we selected LNO as the oxide matrix because of its attractive properties, including ferroelectricity, transparency in the visible range and optical nonlinearity.^34–36^ Furthermore, the LNO matrix can work as the surrounding medium to protect Ag from oxidation. The demonstration of Ag–LNO nanocomposite presents a new approach for the synthesis of Ag and other easily oxidized metal nanostructures.

## Results and discussion

**Fig. 1:**
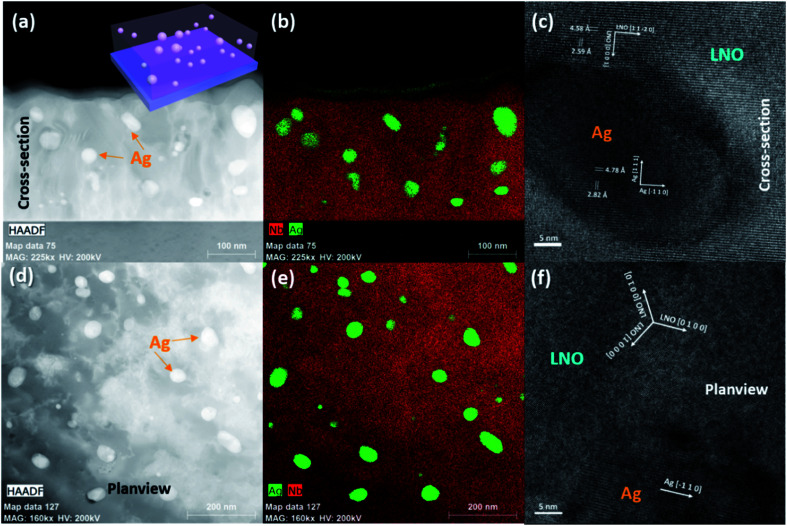
Microstructure characterization of the low-density Ag–LNO nanocomposite thin film. (a) Low-mag cross-sectional STEM image, inset is the schematic and (b) corresponding EDS mapping; (c) high-resolution TEM image to show one representative Ag NP; (d) low-mag plan-view STEM image and (e) corresponding EDS mapping; (f) high-resolutionTEM image to show the Ag/LNO interface.

Ag–LNO with low-density and high-density Ag NPs have been deposited on Al_2_O_3_ (001) using the pulsed laser deposition (PLD) method by controlling the dimension of the Ag strip. Microstructural characterizations have been conducted on both the samples. Fig. 1(a) shows a low-mag scanning transmission electron microscopy (STEM) image with its corresponding energy-dispersive X-ray spectroscopy (EDS) mapping in Fig. 1(b). Low-density Ag NPs with an average diameter of ∼15 nm have been randomly distributed in the LNO matrix. Fig. 1(c) shows a high-mag transmission electron microscopy (TEM) image with a typical Ag NP, which presents high crystallinity in both the Ag and LNO phase. Furthermore, the lattice matching between the Ag NP and LNO could be determined as Ag [1 1 1]/LNO [0 0 0 1] and Ag [−1 1 0]/LNO [1 1 −2 0]. Moreover, a plan-view microstructure is observed in the low-mag STEM image in Fig. 1(d) and its corresponding EDS mapping in Fig. 1(e), which provides the overall 3D look of the low-density Ag–LNO nanocomposite thin film, as illustrated in the schematic in the inset of Fig. 1(a). A high-resolution plan-view TEM image shown in Fig. 1(f) determines the in-plane lattice matching of Ag [−1 1 0]/LNO [0 1 0 0]. Fig. S1† shows the microstructure of the high-density Ag–LNO film, which shows a higher density of the Ag NPs with a larger average size of ∼25 nm compared to the low-density one. Furthermore, X-ray diffraction (XRD) measurements were conducted to characterize the crystallinity of Ag–LNO films compared with the pure LNO film. Only the LNO (0 0 *l*) phase is observed for all these films with or without the embedded Ag NPs, which indicates zero deterioration in the crystallinity of the LNO phase with the introduction of Ag. This could be attributed to the incorporation of Au, thus facilitating the nucleation and growth of LNO, which is very similar to the case of LNO–Au nanocomposite films.^37,38^ For the Ag phase, stronger Ag (1 1 1) and weak Ag (0 0 2) peaks have been determined, which suggests that the majority of Ag follows the (1 1 1) growth direction. Atomic force microscopy (AFM) was performed to detect the surface morphology of Ag–LNO films compared with the pure LNO film. The pure LNO exhibits a very smooth surface with an average roughness of 1.26 nm; moreover, low-density and high-density Ag–LNO films present a higher roughness of 10.2 and 8.06 nm, respectively.

**Fig. 2:**
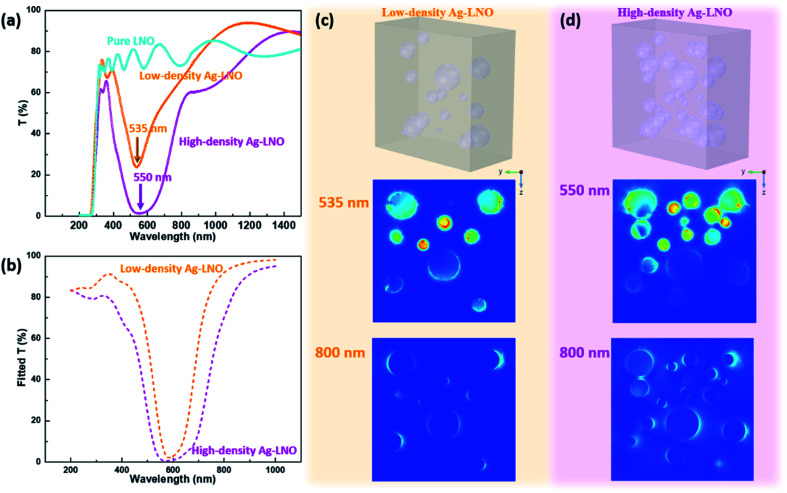
(a) Measured and (b) simulated transmittance of pure LNO, low-density and high-density Ag–LNO thin films; side-view electrical field maps of (c) low-density Ag–LNO at 535 nm and 800 nm and (d) high-density Ag–LNO at 550 nm and 800 nm.

**Fig. 3:**
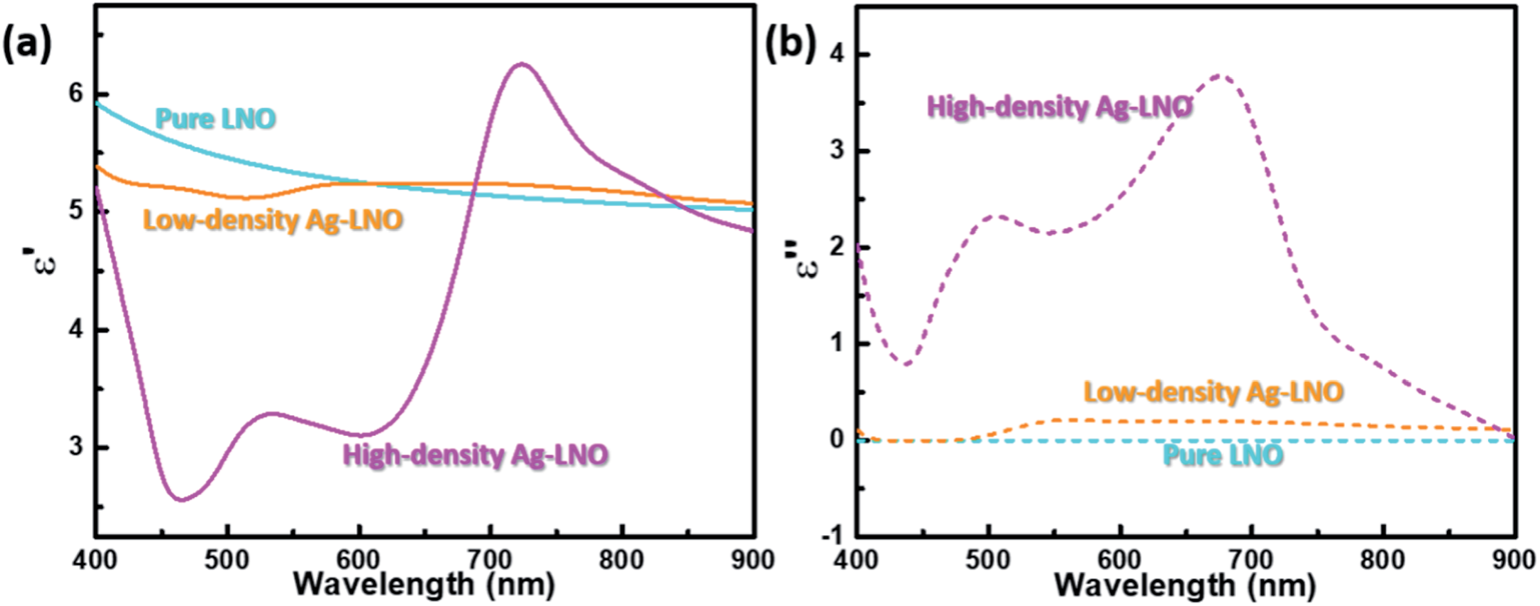
The (a) real and (b) imaginary parts of the complex dielectric function comparison of the pure LNO, low-density and high-density Ag–LNO nanocomposite thin films.

We then focus on the optical performance of the Ag–LNO nanocomposite thin films. First, transmittance measurements at normal incidence were conducted for low-density and high-density Ag–LNO compared with the pure LNO film. The pure LNO shows high transmittance throughout the visible range till 1500 nm. A valley is observed at 535 nm for the low-density Ag–LNO, and a broad valley centered at 550 nm was determined for the high-density Ag–LNO. The transmittance valley corresponds to the surface plasmon resonance (SPR) of Ag NPs, and the different position is attributed to the varying size of the Ag NPs. The red-shift with increase in Ag NPs size follows the spherical particle-in-a-box model proposed by Brus.^39^ Such an SPR position could be reproduced by COMSOL simulation using the approximated geometry of the actual sample (upper panel of Fig. 2(c) for low-density Ag–LNO and Fig. 2(d) for high-density Ag–LNO), as shown in Fig. 2(b). The lower panels of Fig. 3(c) and (d) show the optical field enhancement maps of low-density Ag–LNO at 535 nm, 800 nm and high-density Ag–LNO at 550 nm, 800 nm, respectively. At the SPR position, the resonance is primarily attributed to the strong field enhancement localized at the Ag/LNO interface.

To explore the optical complex dielectric functions of the Ag–LNO nanocomposite thin films, angular-dependent ellipsometry measurements were carried out. The real (*ε*′) and imaginary (*ε*′′) parts of the complex dielectric function of the pure LNO, low-density and high-density Ag–LNO are plotted in Fig. 3(a) and (b), respectively, which were obtained by fitting the measured ellipsometric phi (*φ*) values (shown in Fig. S4†). The refractive index *n* and extinction coefficient *k* of all the samples are compared in Fig. S5.† Compared to the pure LNO film, the incorporation of Ag NPs tunes the optical constants of the entire film, especially the high-density Ag–LNO film. Decreased *ε*′ values are observed in the lower wavelength range, which is attributed to the inclusion of Ag NPs as metals show lower *ε*′ values. However, no epsilon-near-zero (ENZ) is obtained, which might be attributed to the large amount of LNO phase in the film and large *ε*′ values shown by LNO. Furthermore, a broad peak (∼650 nm to ∼800 nm) appears for the high-density Ag–LNO film because of the increased reflection by Ag NPs, which has been revealed in the Au–LNO and Au–TiO_2_ nanocomposite thin film, at different wavelength ranges.^31,40^ Furthermore, the pure LNO exhibits zero *ε*′′ and *k* values because it is transparent in the measured wavelength. The low-density Ag–LNO presents zero *ε*′′ and *k* values until ∼500 nm with an increase toward positive owing to the light absorption by the embedded Ag NPs, which is inconsistent with the above transmittance spectra.

**Fig. 4:**
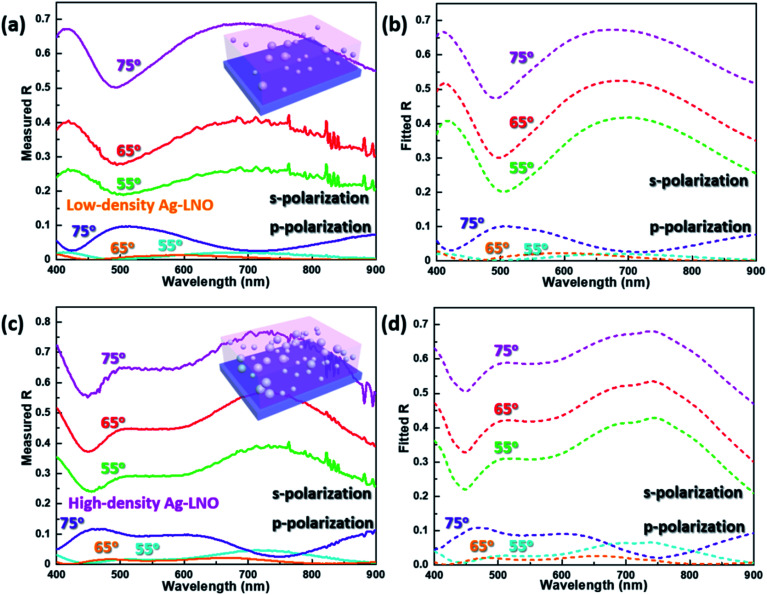
Angle-dependent and polarization-resolved reflectivity (s-polarization and p-polarization) measured at different angles of incidence (55°, 65°, 75°) and corresponding simulated reflectivity spectra. (a) Measured and (b) simulated reflectivity spectra for low-density Ag–LNO; (c) measured and (d) simulated reflectivity spectra for high-density Ag–LNO.

In addition, we employed angle-dependent and polarization-resolved reflectivity measurement (incident angles of 55°, 65°, and 75°) to characterize the optical anisotropy of Ag–LNO films. Fig. 4(a) and (b) show the measured and simulated reflectivity spectral for low-density Ag–LNO, while Fig. 4(c) and (d) present the measured and simulated reflectivity spectral for high-density Ag–LNO. For both these cases, the simulated results reproduced the experimental spectral well. For the s-polarized light incidence (electric field is parallel to the film surface), the reflectivity is higher with larger incident angle, which agrees with the classical electromagnetic theory.^41^ However, considerably different reflectivity spectra are obtained with p-polarized light incidence (electric field is perpendicular to the film surface), which indicates the optical anisotropy of the Ag–LNO nanocomposite thin film.

Overall, the Ag–LNO nanocomposite thin films show interesting optical properties, which can be further tailored by controlling the deposition parameters, such as the target composition, deposition temperature, and laser frequency. This method provides another approach to fabricate Ag nanostructures without oxidation. Moreover, such a Ag–LNO nanocomposite thin film could be integrated on other substrates such as SrTiO_3_ (STO). A typical low-mag STEM image of low-density Ag–LNO is shown in Fig. S6a† with its corresponding EDS mapping presented in Fig. S6b,† which exhibit a similar structure compared to the film on the Al_2_O_3_ substrate. Fig. S6c† shows the transmittance spectra of pure LNO, low-density and high-density Ag–LNO films on STO, which show SPR-related valleys, although the position is slightly changed.

## Conclusion

High-quality Ag–LNO nanocomposite thin films have been deposited, with Ag NPs embedded in LNO matrix. The density and size of Ag NPs were tuned by varying the composition of the target. No crystal quality deterioration was observed for the co-growth of the metal phase Ag and the oxide phase LNO. The Ag–LNO nanocomposite thin films showed interesting optical properties, such as SPR in the visible range, tunable optical constants, as well as optical anisotropy. This work demonstrates an effective approach to synthesize Ag nanoparticles without oxidation, which is promising for future application in photonic devices.

## Experimental

The pure LNO, low-density and high-density Ag–LNO nanocomposite thin films were deposited by pulsed laser deposition (PLD) method with a KrF excimer laser (Lambda Physik, *λ* = 248 nm). The co-deposition of Ag strip and LNO target was applied to deposit the Ag–LNO nanocomposite thin films, the films were obtained by the alternative laser ablation of Ag strips and LNO target. Before the deposition, the base pressure was pumped down to 5 × 10^−7^ torr. During deposition, the temperature was maintained at 650 °C with an O_2_ pressure of 10 mTorr and deposition frequency of 3 Hz. The density of Ag NPs was controlled by the size of the Ag strip. After deposition, the chamber was naturally cooled to room temperature at a rate of 10 °C min^−1^.

The crystal structure and microstructure of films were characterized by XRD (PanalyticalX'Pert X-ray diffractometer) and TEM (FEI Talos-F200X). The AFM images were obtained by Bruker Icon AFM.

Transmittance measurement was then carried out by UV-visible spectroscopy (PerkinElmer Lambda 1050). Optical simulation was conducted by COMSOL Multiphysics Wave Optics Module. Variable angle ellipsometry experiments were then conducted using a RC2 spectroscopic ellipsometer (J.A. Woollam Company). The effective refractive index and optical dielectric constants were obtained by fitting the ellipsometry parameters of psi (*φ*) using different models in VASE.

## Conflicts of interest

There are no conflicts to declare.

## Supplementary Material

NA-003-D0NA00975J-s001

## References

[cit1] MacManus-Driscoll J. L. (2010). Adv. Funct. Mater..

[cit2] Zhang W., Ramesh R., MacManus-Driscoll J. L., Wang H. (2015). MRS Bull..

[cit3] Huang J., MacManus-Driscoll J. L., Wang H. (2017). J. Mater. Res..

[cit4] Chen A., Bi Z., Jia Q., MacManus-Driscoll J. L., Wang H. (2013). Acta Mater..

[cit5] Zheng H., Wang J., Lofland S. E., Ma Z., Mohaddes-Ardabili L., Zhao T., Salamanca-Riba L., Shinde S. R., Ogale S. B., Bai F., Viehland D., Jia Y., Schlom D. G., Wuttig M., Roytburdand A., Ramesh R. (2004). Science.

[cit6] Zavaliche F., Zhao T., Zheng H., Straub F., Cruz M. P., Yang P. L., Hao D., Ramesh R. (2007). Nano Lett..

[cit7] Huang J., Gellatly A., Kauffmann A., Sun X., Wang H. (2018). Cryst. Growth Des..

[cit8] Choi E., Weal E., Bi Z., Wang H., Kursumovic A., Fix T., Blamireand M. G., MacManus-Driscoll J. L. (2013). Appl. Phys. Lett..

[cit9] Fan M., Zhang W., Jian J., Huang J., Wang H. (2016). APL Mater..

[cit10] Harrington S. A., Zhai J., Denev S., Gopalan V., Wang H., Bi Z., Redfern S. A. T., Baek S., Bark C. W., Eom C., Jia Q., Vickers M. E., MacManus-Driscoll J. L. (2011). Nat. Nanotechnol..

[cit11] Lee S., Sangle A., Lu P., Chen A., Zhang W., Lee J., Wang H., Jia Q., MacManus-Driscoll J. L. (2014). Adv. Mater..

[cit12] Chen A., Zhang W., Khatkhatay F., Su Q., Tsai C., Chen L., Jia Q. X., MacManus-Driscoll J. L., Wang H. (2013). Appl. Phys. Lett..

[cit13] Fan M., Thompson M., Andrade M. L., Brolo A. G. (2010). Anal. Chem..

[cit14] Loiseau A., Asila V., Boitel-Aullen G., Lam M., Salmain M., Boujday S. (2019). Biosensors.

[cit15] Tang S., Zheng J. (2018). Adv. Healthcare Mater..

[cit16] Shen W., Tang J., Wang Y., Liu J., Huang L., Chen W., Yang L., Wang W., Wang Y., Yang R., Yun J., Belfiore L. A. (2017). ACS Appl. Mater. Interfaces.

[cit17] Christopher P., Xin H., Linic S. (2011). Nat. Chem..

[cit18] Dong X., Gao Z., Yang K., Zhang W., Xu L. (2015). Catal. Sci. Technol..

[cit19] Zhang Q., Li N., Goebl J., Lu Z., Yin Y. (2011). J. Am. Chem. Soc..

[cit20] Huang L. M., Wang H. T., Wang Z. B., Mitra A., Bozhilov K. N., Yan Y. S. (2002). Adv. Mater..

[cit21] Mafuné F., Kohno J.-y., Takeda Y., Kondowand T., Sawabe H. (2000). J. Phys. Chem. B.

[cit22] Rodriguez-Sanchez L., Blanco M. C., LopezQuintela M. A. (2000). J. Phys. Chem. B.

[cit23] Cobley C. M., Rycenga M., Zhou F., Li Z. Y., Xia Y. (2009). J. Phys. Chem. C.

[cit24] Wang Y., Zheng Y., Huang C. Z., Xia Y. (2013). J. Am. Chem. Soc..

[cit25] Li B., Ye S., Stewart I. E., Alvarez S., Wiley B. J. (2015). Nano Lett..

[cit26] Wu C., Zhou X., Wei J. (2015). Nanoscale Res. Lett..

[cit27] Huang J., Li L., Lu P., Qi Z., Sun X., Zhang X., Wang H. (2017). Nanoscale.

[cit28] Huang J., Qi Z., Li L., Wang H., Xue S., Zhang B., Zhang X., Wang H. (2018). Nanoscale.

[cit29] Li L., Sun L., Gomez-Diaz J. S., Hogan N. L., Lu P., Khatkhatay F., Zhang W., Jian J., Huang J., Su Q., Fan M., Jacob C., Li J., Zhang X., Jia Q., Sheldon M., Alú A., Li X., Wang H. (2016). Nano Lett..

[cit30] Huang J., Wang X., Phuah X. L., Lu P., Qi Z., Wang H. (2019). Materials Today Nano.

[cit31] Huang J., Jin T., Misra S., Wang H., Qi Z., Dai Y., Sun X., Li L., Okkema J., Chen H., Lin P., Zhang X., Wang H. (2018). Adv. Opt. Mater..

[cit32] Jian J., Wang X., Misra S., Sun X., Qi Z., Gao X., Sun J., Donohue A., Lin D. G., Pol V., Youngblood J., Wang H., Li L., Huang J., Wang H. (2019). Adv. Funct. Mater..

[cit33] Qi Z., Jian J., Huang J., Tang J., Wang H., Pol V. G., Wang H. (2018). Nano Energy.

[cit34] Tu D., Xu C. N., Yoshida A., Fujihala M., Hirotsuand J., Zheng X. G. (2017). Adv. Mater..

[cit35] Ievlev A. V., Alikin D. O., Morozovska A. N., Varenyk O. V., Eliseev E. A., Kholkin A. L., Shur V. Y., Kalinin S. V. (2015). ACS Nano.

[cit36] Dutto F., Heiss M., Lovera A., Lopez-Sanchez O., Morral A. F., Radenovic A. (2013). Nano Lett..

[cit37] Ma Z., Xu Y., Allen L. H. (1992). Appl. Phys. Lett..

[cit38] Allen L. H., Phillips J. R., Theodore D., Carter C. B., Soave R., Mayer J. W., Ottaviani G. (1990). Phys. Rev. B: Condens. Matter Mater. Phys..

[cit39] Brus L. (1986). J. Phys. Chem..

[cit40] Misra S., Li L., Jian J., Huang J., Wang X., Zemlyanov D., Jang J., Ribeiro F. H., Wang H. (2018). ACS Appl. Mater. Interfaces.

[cit41] Novotny L. and Hecht B., Principles of nano-optics, Cambridge University Press, 2012

