# Supplementary material for: Hybrid Ag–LiNbO3 nanocomposite thin films with tailorable optical properties
Source: Nanoscale Adv. 2020 Dec 28;3(4):1121–6. doi: 10.1039/d0na00975j (PMC9417351; doi:10.1039/d0na00975j)
Supplement: NA-003-D0NA00975J-s001 [file NA-003-D0NA00975J-s001.pdf]

Electronic Supplementary Material (ESI) for Nanoscale Advances.
This journal is © The Royal Society of Chemistry 2020

Hybrid Ag-$LiNbO_3$ Nanocomposite Thin Films with Tailorable Optical Properties

Jijie Huang[a*], Di Zhang[b], Zhimin Qi[b], Bruce Zhang[b] and Haiyan Wang[bc*]
[a]School of Materials, Sun Yat-sen University, Guangzhou, Guangdong 510275, China
[b]School of Materials Engineering, Purdue University, West Lafayette, IN 47907, USA
[c]School of Electrical and Computer Engineering, Purdue University, West Lafayette, IN 47907, USA
*Correspondence: huangjj83@mail.sysu.edu.cn (J. H.), hwang00@purdue.edu (H. W.).

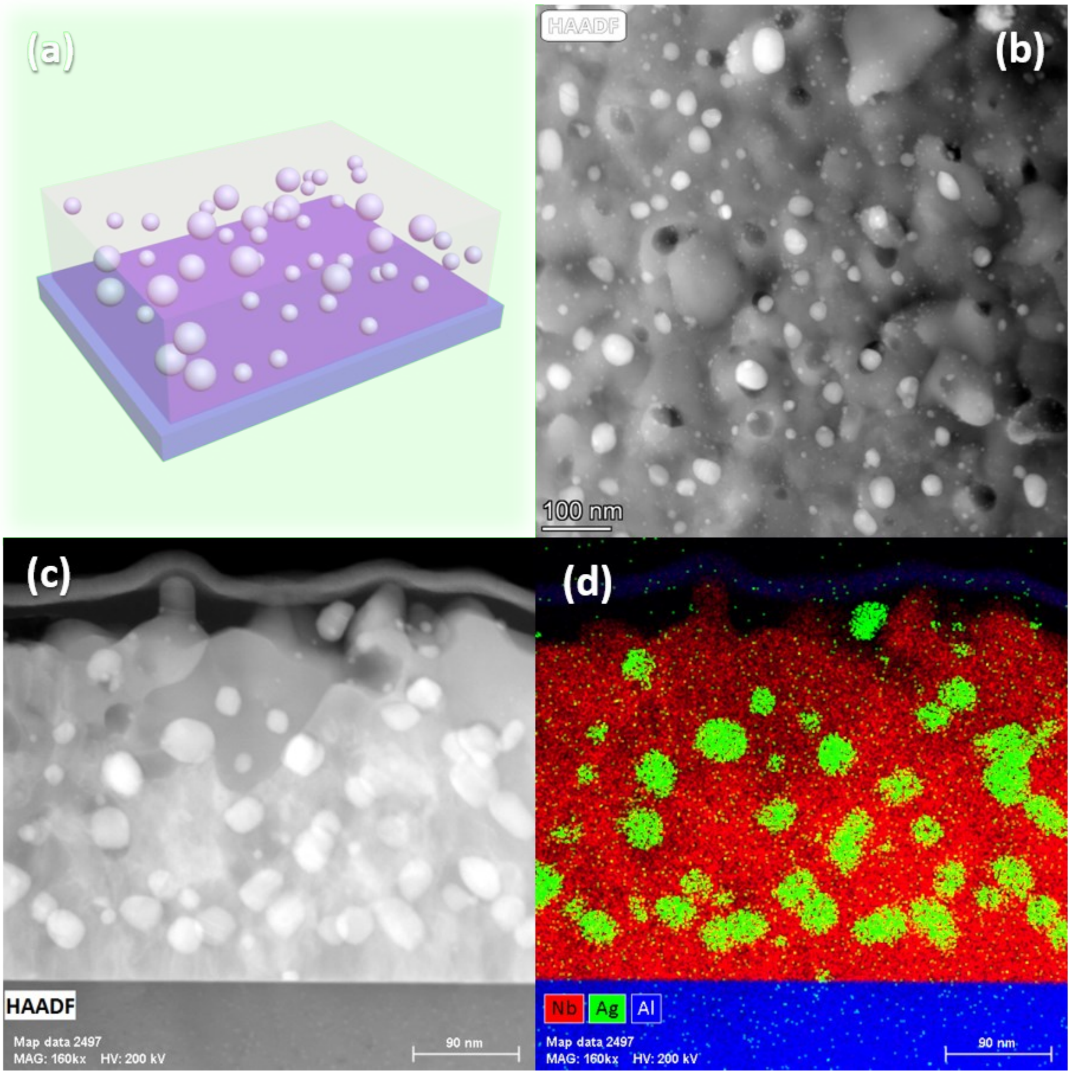

Figure S1. Microstructure characterizations of the high-density Ag-LNO nanocomposite thin film. (a) schematic illustration; (b) low-mag plan-view STEM image; (c) low-mag cross-sectional STEM image and (d) its corresponding EDS mapping.

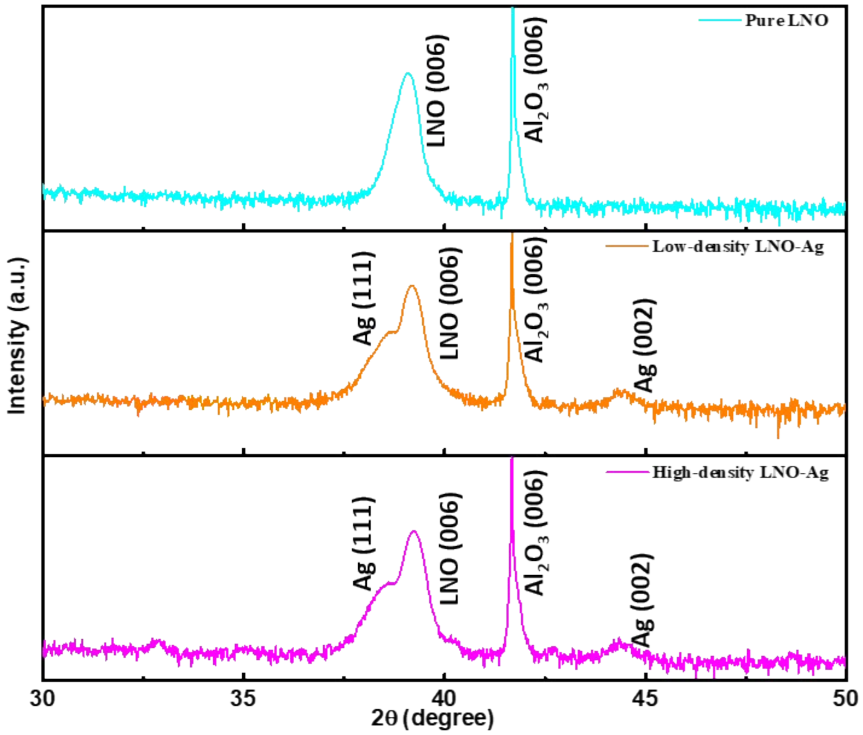

Figure S2. Standard θ-2θ XRD scans of the pure LNO, low-density and high-density Ag-LNO thin films.

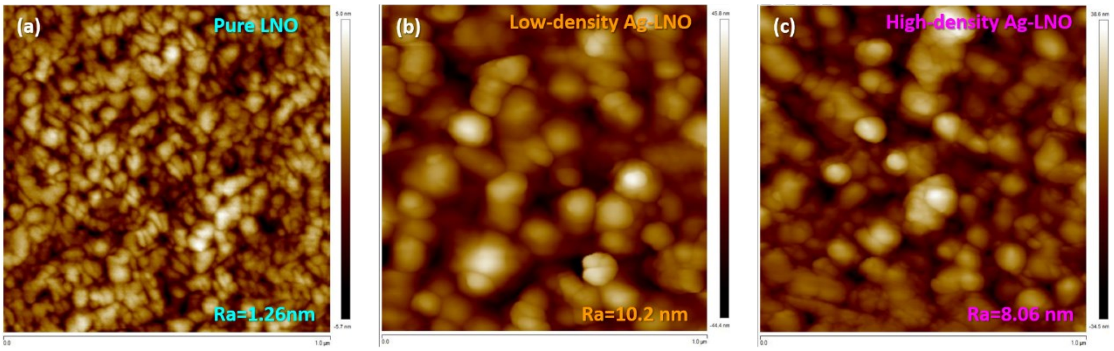

Figure S3. AFM characterizations of (a) the pure LNO, (b) low-density and (c) high-density Ag-LNO thin films.

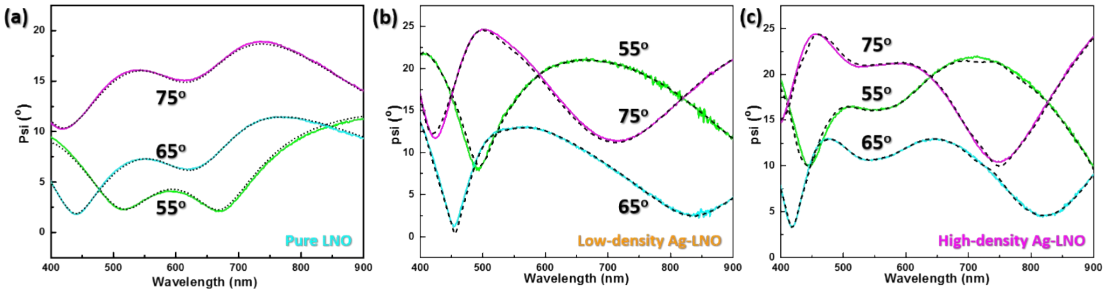

Figure S4. Experimental (solid points) and fitted (solid lines) components at different angles (55°, 65°, 75°) of the ellipsometric parameter Psi (φ) vs. wavelength for (a) pure LNO, (b) low-density and (c) high-density Ag-LNO films.

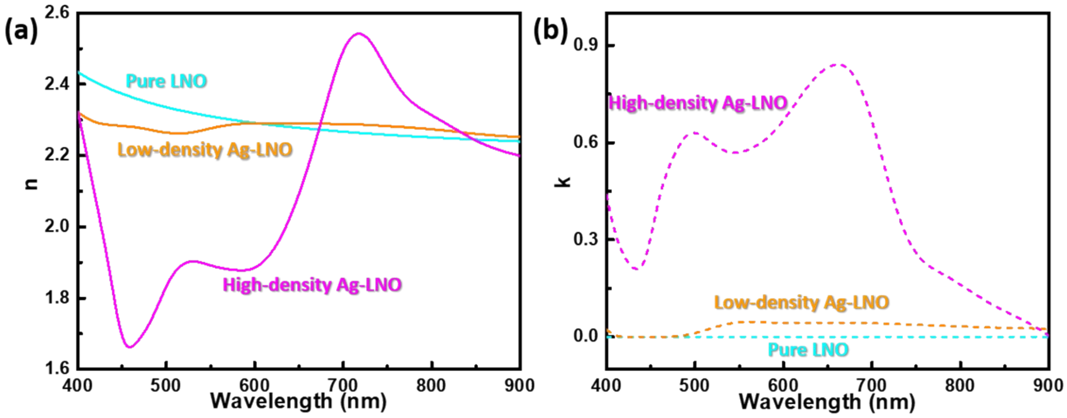

Figure S5. The (a) refractive index *n* and (b) extinction coefficient *k* of the pure LNO, low-density and high-density Ag-LNO nanocomposite thin films.

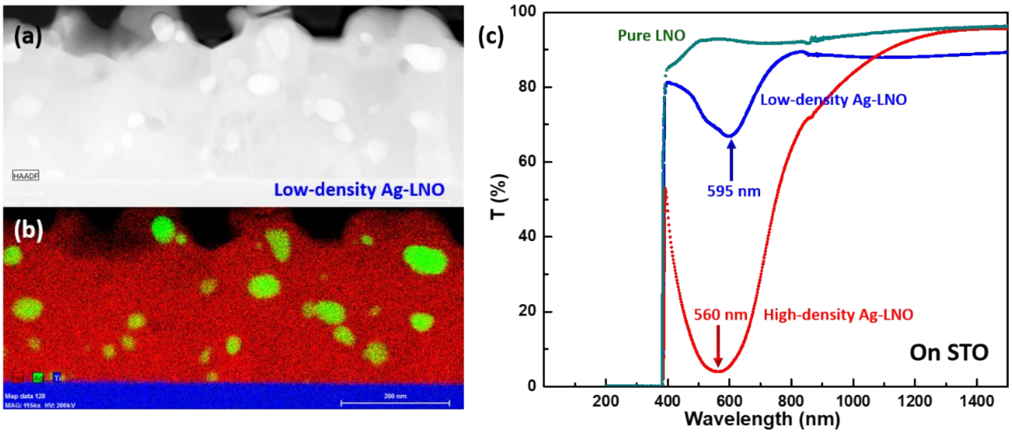

Figure S6. (a) Low-mag STEM image of low-density Ag-LNO grown on STO substrate with (b) corresponding EDS mapping; (c) transmittance spectral of the pure LNO, low-density and high-density Ag-LNO films.
